# IgA-dominant acute poststreptococcal glomerulonephritis with concomitant rheumatic fever successfully treated with steroids: a case report

**DOI:** 10.3325/cmj.2015.56.567

**Published:** 2015-12

**Authors:** Rina R Rus, Nataša Toplak, Alenka Vizjak, Jerica Mraz, Dušan Ferluga

**Affiliations:** 1 Division of Nephrology, University Children’s Hospital Ljubljana, Ljubljana, Slovenia; 2 Department of Allergology, Rheumatology and Clinical Immunology, University Children’s Hospital Ljubljana, Ljubljana, Slovenia; 3 Faculty of Medicine, University of Ljubljana, Ljubljana, Slovenia; 4 Institute of Pathology, Faculty of Medicine Ljubljana, Ljubljana, Slovenia; **Rus et al**: IgA-dominant acute poststreptococcal glomerulonephritis with concomitant rheumatic fever

## Abstract

There are only a few reports of the co-occurrence of acute poststreptococcal glomerulonephritis (APGN) and acute rheumatic fever. We report an unusual case of a 3-year-old boy with nephrotic syndrome and acute renal failure with the transitional need for peritoneal dialysis, biopsy-proven atypical IgA-dominant APGN, and concomitant acute rheumatic fever, successfully treated by steroids. Aggressive treatment with pulses of methylprednisolone proved to be successful and we recommend its use in this type of cases.

Acute poststreptococcal glomerulonephritis (APGN) and acute rheumatic fever (ARF) are well-known nonsuppurative complications of group A streptococcus infection, but they rarely co-occur in the same child (1-3). The cases in children were mostly treated by furosemide and salicylate, and benzathine penicillin prophylaxis, and one case was successfully treated by oral prednisolone (2).

To the best of our knowledge, this is the first case of IgA-dominant APGN manifesting as acute kidney injury due to a rapidly progressive glomerulonephritis, consequent renal failure, and the need for transitional peritoneal dialysis, co-occurring with ARF in a small child, which was successfully treated with pulse methylprednisolone therapy.

## Case report

The patient was born at 40 weeks of gestation after an uneventful pregnancy. He had a rotavirus infection at nine months and urinary tract infection at the age of two years. His mother had a history of mild asthma and pollen allergy. Informed consent for writing of this case report was obtained from patient’s parents.

### Patient history

The 3-year-old boy became febrile four days before admission to the hospital. He had dark urine, complained of pain in his right knee, and started to limp. His first laboratory findings showed an increase in C-reactive protein (32 mg/L, N<8), leukocytes 12.7 × 10^9^/L, blood urea nitrogen (BUN) 15.9 mmol/L (N 2.8-7.5), and creatinine 115 umol/L (N 44-97). Urine analysis showed proteinuria (dipstick 3+) and blood (3+) in the urine. Three weeks before admission, he had acute tonsillopharyngitis with fever, which resolved spontaneously without antibiotic treatment. A few days before his episode of acute tonsillopharyngitis, his mother had the same symptoms and was treated with penicillin.

### Clinical findings, diagnostic assessment, and the course of treatment

On admission, he had signs of mild respiratory infection with a hyperemic pharynx. He had no signs of edema. Blood pressure was normal (107/45 mm Hg), as well as auscultation of the lungs and heart. He was febrile during the first two days of hospitalization, and showed signs of nephritic syndrome (oliguria, azotemia with increased creatinine, hematuria, and proteinuria). During hospitalization, his blood pressure remained normal. The erythrocyte sedimentation rate (56 mm/h) was increased. He developed signs of nephrotic syndrome (hypoproteinemia 48 g/L [N 65-80], hypoalbuminemia 27 g/L [N 32-55], hyperlipemia [cholesterol 7.5, N 4.0-5.2]), proteinuria increased to nephrotic range (90 mg/h/m^2^), and hematuria persisted (1707 erythrocytes/high power field).

On the second day of hospitalization, a systolic heart murmur 3/6 appeared. Echocardiography showed minimal pericardial effusion, with mild to moderate mitral regurgitation and minimal aortic regurgitation. During hospitalization, the boy became edematous, with ascites. Chest x-ray showed pleural effusion with mild pulmonary interstitial congestion and the patient became anuric and gained 2.7 kg despite continuous furosemide infusion (maximally 1 mg/kg/h) administered since the first day of admission. The highest BUN value was 27 mmol/L and the highest creatinine value 240 umol/L. Metabolic acidosis was observed. Potassium, chloride, sodium, and magnesium were within the reference ranges and phosphate was transitionally elevated (2.29 mmol/L, N 0.8-1.4). Due to hypervolemia, an acute peritoneal catheter was inserted. Peritoneal dialysis was started on the sixth day of hospitalization and was continued for 10 days. Two weeks after the start of the disease, skin peeling on the palms and feet was observed.

**Figure 1:**
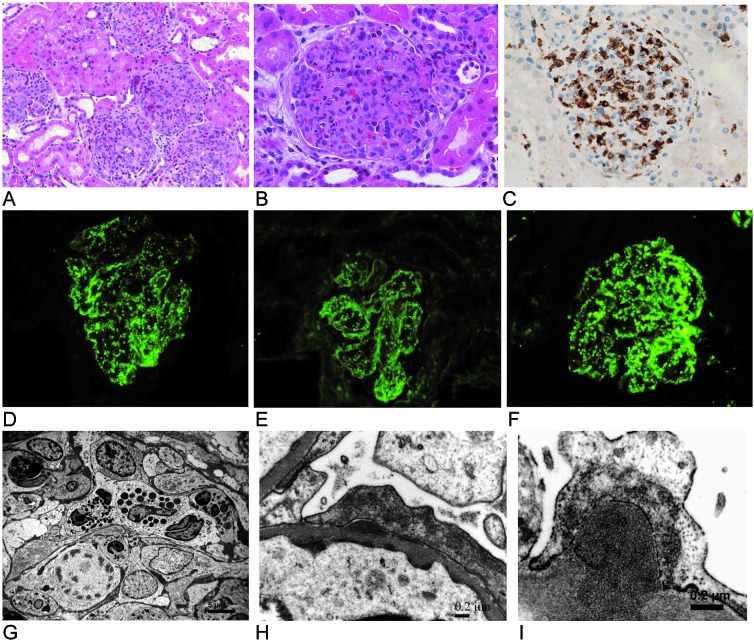
(**A**) Five enlarged glomeruli in IgA-dominant acute poststreptococcal glomerulonephritis showing diffuse global endocapillary hypercellularity (hematoxylin and eosin [HE] staining). (**B**) An enlarged glomerulus with global closure of the capillary lumina caused by endogeneous predominantly mesangial cell proliferation and infiltrating blood-borne monocytes and polymorphonuclear leukocytes (HE staining). (**C**) Numerous brown stained CD68-positive monocytes infiltrating the enlarged hypercellular glomerular tuft (anti-CD68 monoclonal antibody, clone KP-1, immunohistochemistry using ultraView DAB detection kit, Ventana Medical Systems, Tuscon, AZ, USA). (**D-F**) Immunofluorescence microscopy showing granular mesangial and glomerular capillary wall immune deposits with a “starry-sky” pattern of IgA dominance (**D**), less intensive IgG (**E**) and prominent C3 (**F**). (**G**) Part of a glomerular lobule showing a pronounced endocapillary hypercellularity caused by proliferating (note one mitotic figure) predominant mesangial cells, surrounded by a mesangial matrix with discrete dense deposits, as well as by infiltrating leukocytes (electron micrograph). (**H**) Discrete electron dense subendothelial and subepithelial deposits detected using high magnification (electron micrograph). (**I**) A solitary hump-shaped electron dense deposit on the outer aspect of the glomerular basement membrane (electron micrograph).

Additional laboratory tests showed anemia; hemoglobin decreased from 112 g/L to 70 g/L, as well as increased anti-streptolysin O titer (441 IU/mL, N<170), but the throat swab culture and the blood culture remained negative. The classical (46%, N 72-128) and alternative (38 IU, N 80-120) complement pathways and C3 level were decreased (696 mg/L, N 970-1576), while the C4 level was within the reference range. Antinuclear antigen antibodies, anti-DNA, anti-beta 2 glycoprotein antibodies, and anticardiolipin antibodies were negative. No genetic tests were performed. Ultrasonography showed enlarged and hyperechogenic kidneys. Due to nephrotic-nephritic syndrome with a clinical course of rapidly progressive glomerulonephritis, renal biopsy was performed. The biopsy showed severe diffuse global endocapillary proliferative glomerulonephritis with moderate intensity exudation of neutrophils and macrophages. Fifteen percent of glomeruli exhibited extracapillary crescents. Immunofluorescence showed a “starry sky” pattern of granular mesangial and glomerular capillary wall immune deposits positive for IgA 2+, IgG 1+, C3 4+, C4 1+ and fibrin/fibrinogen 1+. C1q was negative. Electron microscopy showed irregular mesangial and subendothelial electron-dense deposits associated with a few small subepithelial hump-shaped deposits (Figure 1). Renal biopsy findings, clinical picture, and laboratory results suggested a diagnosis of IgA-dominant APGN. On the basis of clinical picture, biopsy examinations, laboratory work-up, and imaging analysis, we concluded that acute kidney injury with renal failure had to be ascribed to APGN. This diagnosis was accompanied by concomitant ARF with carditis as the major diagnostic criterion, and fever, elevated acute phase reactants, and arthralgia as the minor criteria (4). We started penicillin V treatment (75 mg/kg/d in 3 divided doses), followed by penicillin prophylaxis (penicillin G benzathine 600 000 units as a single dose) with intramuscular injections every four weeks. Methylprednisolone pulse therapy was started on the seventh day after hospital admission. After three pulses (10 mg/kg), the dose was slowly tapered over the next three months.

*Follow up and outcome.* At the follow-up visit after two months, the patient’s general condition and renal function were improved, anemia subsided, and the acute phase reactants, anti-streptolysin O titer, and complement levels returned to reference ranges, and microhematuria persisted. Heart and kidney ultrasonography were normal. At the follow-up visit after four years, there were no renal or cardiac sequels (Table 1).

**Table 1:** Timeline

Beginning of February 2010	Acute tonsilopharingitis
February 23, 2010	Febrile, dark urine, pain in the right knee – limping, slight increase in creatinine
February 27, 2010 - admission to hospital	Mild respiratory infection, auscultation of heart and lungs normal, nephritic syndrome with increased creatinine, oliguria, no edema
2nd day of hospitalization - 2010	Heart murmur – echocardiography with pathological findings
6th day of hospitalization – 2010	Edema, ascites, hypervolemia, anuria, peritoneal dialysis (PD), and methylprednisolone pulses were started, renal biopsy was performed
16th day of hospitalization – 2010	PD catheter was removed, diuresis was restored, creatinine decreased, complement low
May 2010	Patient’s general condition improved, renal function and complement were normal, microhematuria persisted
2014-2015	No cardiac or renal sequels

## Discussion

A rapidly progressive course of glomerulonephritis with acute renal failure rarely occurs in small children. Its most frequently reported rare causes are APGN, Henoch-Schönlein purpura nephritis, IgA nephropathy, and lupus nephritis (5). Given the clinical picture and laboratory tests, the three latter causes were less likely to have been the causes in our case. Therefore, the most likely cause of renal disease was APGN, and such assumption was also supported by the occurrence of acute tonsilopharingitis three weeks before renal disease, hypocomplementemia with low C3 level, increased anti-streptolysin O titer, and negative tests for systemic lupus erythematosus. Renal biopsy confirmed the diagnosis of atypical IgA APGN, accompanied by ARF confirmed by major and minor diagnostic criteria (4).

The coexistence of APGN and ARF rarely occurs, but it has been previously described (1-3,6,7). The explanation for the co-occurrence might be that certain strains have nephritogenic and rheumatogenic potential, which can result in the occurrence of both sequels (6,8).

The pathogenesis of ARF is well known. The disease is caused by the mechanism of molecular mimicry. Some streptococcal antigens, such as M protein and hyaluronate capsule, have identical antigen epitopes as human tissue proteins in the myocardium, brain, and joints. In this way they cause inflammation by inducing cross reacting autoantibodies against human tissue proteins stimulated by cell mediated immunity in genetically predisposed patients (9). M protein can also stimulate an autoimmune response of a host by playing the role of superantigen.

The immunopathogenesis of APGN is not yet completely understood and the most extensively studied mechanisms are circulating immune complex deposition, *in situ* immune complex formation, and molecular mimicry between streptococcal and human glomerular proteins, with consequent autoimmune response (10). There are probably different pathways leading to glomerular injury (10). It seems that the process is initiated by the glomerular plasmin-binding activity of streptococcal glyceraldehid-3-phosphate dehydrogenase, inducing inflammatory reaction and glomerular basement membrane injury. This enables *in situ* formation of immune complexes, as well as deposition of circulating immune complexes, containing cationic streptococcal antigens, such as streptococcal pyrogenic exotoxin B, which was found to co-localize with complement and IgG in glomerular subepithelial deposits. Complement activation by alternative, classical, and lectin pathways seems also to be an important feature of APGN. Anti-IgG antibodies that bind to IgG Fc receptors in the streptococcal wall, have been found in APGN but do not seem to play an important role in its pathogenesis (10). Although both APGN and ARF are associated with streptococcal infection, they have different pathogenesis so it is not surprising that they co-occur so rarely.

APGN is a classic form of acute postinfectious glomerulonephritis, characterized by nephritic syndrome occurring mostly after pharyngeal acute infection, hypocomplementemia, and diffuse endoproliferative and exudative glomerulonephritis associated with C3-dominant immunofluorescence and hump-shaped subepithelial electron-dense deposits (11). Over recent decades, the spectrum of the disease has changed (12). A number of reports have presented cases of atypical acute postinfectious glomerulonephritis, particularly following staphylococcal infection, shown by immunofluorescence IgA-dominant glomerular staining, endocapillary proliferative pattern, diffuse mesangioproliferative or even focal proliferative histomorphological patterns, and occasionally the absence of hump-shaped deposits (13,14). In our case, biopsy demonstrated a classic histomorphology typical of APGN. However, an unusual finding was that the predominant immunoglobulin in the overall immunoglobulin profile was IgA, similar to an observation from a previous study (15).

The treatment of ARF includes antibiotic therapy, regardless of whether pharyngitis is present at the time of the diagnosis (16). Our patient was also treated with penicillin. Due to the rapidly progressive clinical course of IgA-dominant APGN with concomitant nephrotic syndrome, we decided to treat the child aggressively with methylprednisolone pulses. Patients with APGN complicated by nephrotic syndrome have poor prognosis, with a higher possibility of chronic renal disease (17). Also, ARF can be treated by glucocorticoid therapy (18). We decided against using aspirin due to its potential undesirable side effects on kidney function (19), which was already seriously deteriorated. After treatment with corticosteroids, renal function improved, nephritis syndrome and carditis subsided, and the child completely recovered after 2 months, with the exception of microhematuria.

In conclusion, this is the first case of IgA-dominant APGN manifesting as acute kidney injury due to rapidly progressive glomerulonephritis occurring in a small child at the same time as ARF. Aggressive treatment with pulses of methylprednisolone proved to be successful and we recommend its use in this type of cases.
